# Real-world Implementation of a Smartphone-Based Psychoeducation Program for Bipolar Disorder: Observational Ecological Study

**DOI:** 10.2196/31565

**Published:** 2022-02-02

**Authors:** Aitana García-Estela, Jordi Cantillo, Natalia Angarita-Osorio, Estanislao Mur-Milà, Gerard Anmella, Víctor Pérez, Eduard Vieta, Diego Hidalgo-Mazzei, Francesc Colom

**Affiliations:** 1 Mental Health Research Group Hospital del Mar Medical Research Institute (IMIM) Barcelona Spain; 2 Department of Psychiatry and Forensic Medicine School of Medicine Universitat Autònoma de Barcelona Barcelona Spain; 3 Institute of Neuropsychiatry and Addictions Hospital del Mar Parc de Salut Mar Barcelona Spain; 4 Bipolar and Depressive Disorders Unit Department of Psychiatry and Psychology Institute of Neuroscience, Hospital Clínic, Universitat de Barcelona Barcelona Spain; 5 Centre for Biomedical Research in Mental Health Network (CIBERSAM) Madrid Spain; 6 Centre for Affective Disorders Institute of Psychiatry, Psychology & Neuroscience King's College London London United Kingdom; 7 Department of Basic, Evolutive and Education Psychology Faculty of Psychology Universitat Autònoma de Barcelona Barcelona Spain

**Keywords:** bipolar disorder, psychoeducation, smartphone, app, SIMPLe, Intervention, mobile phone

## Abstract

**Background:**

SIMPLe is an internet‐delivered self‐management mobile app for bipolar disorder (BD) designed to combine technology with evidence-based interventions and facilitate access to psychoeducational content. The SIMPLe app was launched to the real world to make it available worldwide within the context of BD treatment.

**Objective:**

The main aims of this study are as follows: to describe app use, engagement, and retention rates based on server data; to identify patterns of user retention over the first 6-month follow-up of use; and to explore potential factors contributing to discontinuation of app use.

**Methods:**

This was an observational ecological study in which we pooled available data from a real-world implementation of the SIMPLe app. Participation was open on the project website, and the data-collection sources were a web-based questionnaire on clinical data and treatment history administered at inclusion and at 6 months, subjective data gathered through continuous app use, and the use patterns captured by the app server. Characteristics and engagement of regular users, occasional users, and no users were compared using 2-tailed *t* tests or analysis of variance or their nonparametric equivalent. Survival analysis and risk functions were applied to regular users’ data to examine and compare use and user retention. In addition, a user evaluation analysis was performed based on satisfaction, perceived usefulness, and reasons to discontinue app use.

**Results:**

We included 503 participants with data collected between 2016 and 2018, of whom 77.5% (n=390) used the app. Among the app users, 44.4% (173/390) completed the follow-up assessment, and data from these participants were used in our analyses. Engagement declined gradually over the first 6 months of use. The probability of retention of the regular users after 1 month of app use was 67.4% (263/390; 95% CI 62.7%-72.4%). Age (*P*=.002), time passed since illness onset (*P*<.001), and years since diagnosis of BD (*P*=.048) correlate with retention duration. In addition, participants who had been diagnosed with BD for longer used the app on more days (mean 97.73, SD 69.15 days; *P*=.002) than those who had had a more recent onset (mean 66.49, SD 66.18 days; *P*=.002) or those who had been diagnosed more recently (mean 73.45, SD 66 days; *P*=.01).

**Conclusions:**

The user retention rate of the app decreased rapidly after each month until reaching only one-third of the users at 6 months. There exists a strong association between age and app engagement of individuals with BD. Other variables such as years lived with BD, diagnosis of an anxiety disorder, and taking antipsychotics seem relevant as well. Understanding these associations can help in the definition of the most suitable user profiles for predicting trends of engagement, optimization of app prescription, and management.

## Introduction

### Background

Globally, an estimated 46 million people have been diagnosed with bipolar disorder (BD) [1]. Besides behavioral changes occurring during mood episodes, BD has a serious impact on psychosocial functioning, cognition, quality of life, and survival rate of individuals with this condition [2,3]. Although ranges vary dramatically because of methodological differences among studies, there is agreement that people with BD are between 9 and 30 times more likely to die from suicide than someone without this condition [4,5].

Although the fundamental treatment for BD relies basically on psychopharmacology, some adjuvant psychological interventions have been shown to improve the long-term outcomes of this disease [6]. Among psychological interventions, psychoeducation programs have proven to be a cost-effective approach to prevent episodes by helping patients to improve adherence, embrace healthy habits, and learn to recognize the prodromes and symptoms of upcoming episodes. It is obvious that efficacious treatments only work for patients who can receive them [7]; yet, these psychological interventions are scarce and difficult to access for most patients [8].

e-Mental health—the delivery of mental health–related tools through the internet and related technologies [9]—is one of the most promising strategies to address this access gap, relieving overburdened mental health services and increasing the services’ cost-efficiency while maintaining its quality. Although internet-based interventions for mental health are still relatively new, the number of solutions such as web-based platforms, smartphone apps, and wearables is rapidly growing globally [10], with their actual acceptability by patients tending to be increasingly higher [11].

However, the strong desire to proliferate e-mental health solutions has not been translated into a transformation in the delivery of mental health care because there is little available evidence of uptake of mental health apps [12]. Moreover, despite the increasing number of e-mental health apps available in app stores, few are properly validated in a scientific process; this lack of validation could jeopardize the safety and health of potential users [13,14]. In addition, the quality of the content of the apps for BD in the app stores does not live up to expectations because most of them do not rely on the available best practice clinical guidelines [15]. Thus, it remains a challenge to implement platforms developed within evidence-based–practice frames that, simultaneously, have been subjected to efficacy tests [16,17].

Within this framework, the SIMPLe project was designed to leverage the potential of combining technology with evidence-based interventions by developing and evaluating an internet‐delivered self‐management mobile app (SIMPLe 1.5) for BD in addition to standard treatment. The SIMPLe 1.5 app collects information about potential symptoms, with the advantage of providing users with personalized psychoeducational messages and alerts that are tailored to specific needs. The app is based on group psychoeducation, a well-established and evidence-based care-focused psychological intervention that addresses relevant issues of self-management for BD, such as identification and management of early warning signs, lifestyle, and treatment adherence [18,19].

Up until now, the SIMPLe app has proved acceptable to users and has shown interesting and optimistic results: a high retention rate was attained in a 3-month feasibility study and positive outcomes regarding satisfaction were found in a naturalistic implementation feasibility study [20-22]. In addition, some potential improvements in terms of biological rhythms and medication adherence were suggested by post hoc analyses [23]. It is worth mentioning that pharmacological treatment adherence is a particularly complex issue in BD because more than 50% of the patients are estimated to be nonadherent fully to the prescribed doses of medication [24].

Considering that the ultimate aim of the SIMPLe project is to extend and facilitate access to psychoeducational content through the SIMPLe app to all potential users, wide and free access to the SIMPLe 1.5 app around the Spanish-speaking world was offered. This way, we had the opportunity to routinely collect implementation data on use in a real-world setting and naturalistic condition.

A previous report on the OpenSIMPLe study presented partial data demonstrating the high dropout rates when a psychoeducation smartphone-based intervention for individuals with BD is offered openly [22]. We hereby present the results of the whole sample of SIMPLe users throughout the study. This research differs from our previous partial sample in terms of both sample size and follow-up time frame. Statistical analyses of the previous partial sample only included descriptive, pre–post, and logistic regression tests without considering the temporal retention period as we did in this paper.

In this paper, we focus on exploratory analyses that aim to investigate in depth the relationships among variables that may predict overall engagement as well as retention rates mainly by means of survival analyses. More specifically, we intend to shed some light on the ways in which the SIMPLe app engagement and user retention patterns are influenced by individual variables, including sociodemographic and clinical data.

This study is based on an ecological experimental implementation of an e-mental health resource. Thus, the reader may miss the usual randomized controlled studies’ constraints such as the lack of sample size calculation (which, by definition, would be *the bigger the better*) or some control measures. However, this study reflects the real day-to-day problems faced by a mental health app when launched to the app stores to be used by the target population.

### Objectives

The main aims of this study are to (1) describe app use, engagement, and retention rates based on server data; (2) identify patterns of user retention over the first 6-month follow-up of use; and (3) explore potential factors contributing to the risk of discontinuing app use.

The expectations are that these exploratory analyses will help to confirm preliminary use data of the SIMPLe app and understand user retention rates as well as the ways in which users self-manage BD in a real-world setting. In addition, we hope that the results will provide our research colleagues with relevant insights into the interplay, dynamics, and predictive factors of user engagement with mental health apps at the time of their implementation in real-world conditions.

## Methods

### Procedure and Participants

Participation availability was open on the project website [25] to anyone with BD who was fluent in Spanish. Specific app characteristics and functionality offered for the SIMPLe app have been published elsewhere [20,21]. In brief, the app consisted of a daily graphic 5-item screening test (mood, energy, sleep time, medication adherence, and irritability) and a weekly, more comprehensive Yes or No test, considering all Diagnostic and Statistical Manual of Mental Disorders, Fifth Edition, criteria for manic and depressive episodes. The resulting mood assessments were displayed in a chart on the home screen. On the basis of the information collected, a daily pop-up notification prompted the user to read a short psychoeducational message providing brief information or advice about how to deal with specific situations to avoid relapses. Each message was extracted from a library of more than 500 messages categorized according to different clinical situations based on a published psychoeducation manual and lay-language books on BD written by 2 of the authors (FC and EV). Additional optional modules available in the app were personalized medication reminders, prodromal symptoms, and mood-chart sharing.

Following a real-world naturalistic approach, no active recruitment strategy or advertisement was used. Potential participants approached the study voluntarily through the website [25] (more detailed information can be found elsewhere [22]). Potential participants needed to meet the following inclusion criteria: (1) aged ≥18 years; (2) having a psychiatrist-confirmed diagnosis of BD before entry; (3) pharmacological treatment for BD provided by a psychiatrist; (4) owning and using daily an Android or iOS smartphone; (5) fluency in Spanish; (6) an active email account; and (7) standard cutoff score of 7, co-occurrence of at least two symptoms, and moderate or severe impairment on a modified version of the Mood Disorder Questionnaire (MDQ) [26,27]. The participants’ answers were reviewed by a psychiatrist to assess consistency, after which they were informed whether they were eligible for the study.

To prevent duplicate use and potential misuse, the possibility of completing the questionnaire multiple times from the same IP address was blocked. Web-based technical support was provided to the app users, if needed, through email.

Data from the sample were drawn from 503 SIMPLe users who had provided informed consent and completed the app’s onboarding questionnaire between May 2016 and June 2018. Of the 503 participants, 390 (77.5%) used the app, and data from these participants were used in the analyses.

All procedures contributing to this work complied with the ethical standards of national and international guidelines and the basic principles of protection of dignity and human rights, as stated in the Declaration of Helsinki (64th General Assembly, Fortaleza, Brazil, October 2013), and were conducted according to current regulations. The ethical committees from both Hospital Clínic of Barcelona (HCB/2016/0403) and the Hospital del Mar Medical Research Institute (2016/6764/I) approved the protocol.

### Assessments

In all, two sources of data were used: (1) a web-based form administered at program inclusion and at 6 months and (2) subjective data gathered through continuous app use and the use patterns captured by the app server.

#### Psychometric External Assessments

Sociodemographic data as well as illness and treatment history were collected at program inclusion through a web-based form from participants who had provided informed consent. The number of hospitalizations and suicide attempts as well as treatment history during the past 6 months were also collected 6 months after inclusion.

The Spanish validated version of the 5-item World Health Organization Well-being Index [28] was used to assess mental well-being at baseline and 6 months later at the final follow-up.

#### App-Derived Assessments

##### Subjective Information

The subjective information assessed was as follows:

1. Self-reported mood, sleep, medication adherence, and energy: The app prompts users to answer 5 daily slider screening tests on mood, energy, sleep time, irritability, and drug-treatment adherence. The daily scores appearing in the chart are the results of an algorithm, which was previously tested during the development phase [20]. In addition, a more comprehensive test, considering Diagnostic and Statistical Manual of Mental Disorders, Fifth Edition, criteria for manic and depressive episodes, including suicide thoughts, was prompted weekly.
2. Self-reported usability: The app was evaluated by users who made it through to complete follow-up assessment. The users’ perception of the usability of the app was measured through the System Usability Scale (SUS) [29], a 10-item questionnaire with 5 response options (from
  strongly agree to strongly disagree) that allows evaluation of products and services. Interpretation of the raw scores was achieved by converting them into percentile ranks [30] and associating them with adjectives [31].
3. Satisfaction and perceived helpfulness: Satisfaction and perceived helpfulness of each subcomponent of the app were assessed in the follow-up questionnaire through Likert scales after 6 months of program initiation. Suggestions and comments regarding the app were also registered.

##### Mobile Terminal–Mined Information

User retention, app use, and engagement data were constantly recorded at the servers over the study duration, reflecting continuously and in detail app use and engagement. User retention was defined as the proportion of participants who used the app for the entire duration of the study and completed the 6-month follow-up assessment. The SIMPLe app has multiple components, three of which we consider the core active ingredients: the daily and weekly tests and the psychoeducational messages. To determine retention, we considered the user to be active if we registered data in the servers from these 3 interactions, and we considered the user to have discontinued participation if there was a lack of data from these variables in the server for >1 month.

Engagement with mobile apps is considered a multidimensional construct, and different definitions can be used or combined to measure it. In this study, engagement was understood as the ability of an app to engage users and sustain user interactions and it was assessed through indicators such as usability, acceptability, and feasibility [32]. In this case, engagement was calculated based on the weekly percentage of completed tasks (ie, answering daily and weekly tests and reading the daily psychoeducational messages).

### Design

This is an observational ecological study in which we pooled available data from a real-world implementation of the SIMPLe app.

The rationale for the OpenSIMPLe study and detailed methods have been published elsewhere [22]; the methods are briefly outlined here.

### Statistical Analysis

Smartphone app data (ie, participants’ mood ratings, manic or hypomanic and depressive symptoms, and details of their use of the app) were downloaded directly from the servers. Likewise, the users’ baseline and follow-up responses at both baseline and follow-up web-based questionnaires were retrieved from the servers. All analyses were run using SPSS software (version 26.0; IBM Corp) and R statistical package for Windows (version 4.0.2; The R Foundation for Statistical Computing).

Initially, basic descriptive statistics of sociodemographic and clinical variables were run, including age, sex, marital status, family psychiatric history, follow-up time, number of episodes, substance abuse, and comorbid medical and psychiatric diagnoses. Continuous variables have been described based on the mean and SD; the median and the 25th and 75th percentiles have also been used in comparative analyses of the time spent using the SIMPLe 1.5 app. We defined categorical variables in terms of the number and percentage of users per response category. In addition, statistical techniques were used to confirm assumptions of the statistical tests before carrying out parametric tests to compare means and proportions. When the assumptions of parametric tests were violated, nonparametric tests were used.

We performed a comparative analysis of the variables among groups using a 2-tailed *t* test or analysis of variance on continuous variables or their nonparametric equivalent; the Wilcoxon test or the Kruskal–Wallis test was performed depending on the inherent characteristics of the variables analyzed; the Mann–Whitney U test was carried out when dependent variables were ordinal; and the chi-square test was performed when analyzing categorical variables.

The Spearman correlation coefficient (*ρ*) was used to examine correlations between SIMPLe 1.5 app time use and the different continuous variables.

A subsample of users with *engagement* ≥12% was selected for the analysis of the use time of the SIMPLe 1.5 app to determine a minimum frequency of use and avoid overestimating the use time. A homogeneity analysis was performed between the selected subsample and the rest of the participants using the chi-square test for categorical variables and the Kolmogorov–Smirnov Z test for continuous variables.

Survival analysis was used on the selected subsample to examine the time spent using the SIMPLe 1.5 app because the length of the follow-up period was variable and there were participants who did not experience the event *quit using the app* during the 6-month follow-up. Estimates of survival and risk functions of the time use of the app were calculated by applying the Kaplan–Meier method. We used the log-rank test to compare various survival distributions and the Cox proportional-hazards model validated by Schoenfeld residual analysis to assess risks related to the survival of the app users. A sensitivity analysis was performed by repeating the survival analysis with all users to evaluate the effect of the selection of the subsample in the study of the use time. Results were considered significant with 2-sided *P* values <.05.

## Results

### Descriptive Analysis

**Figure 1:**
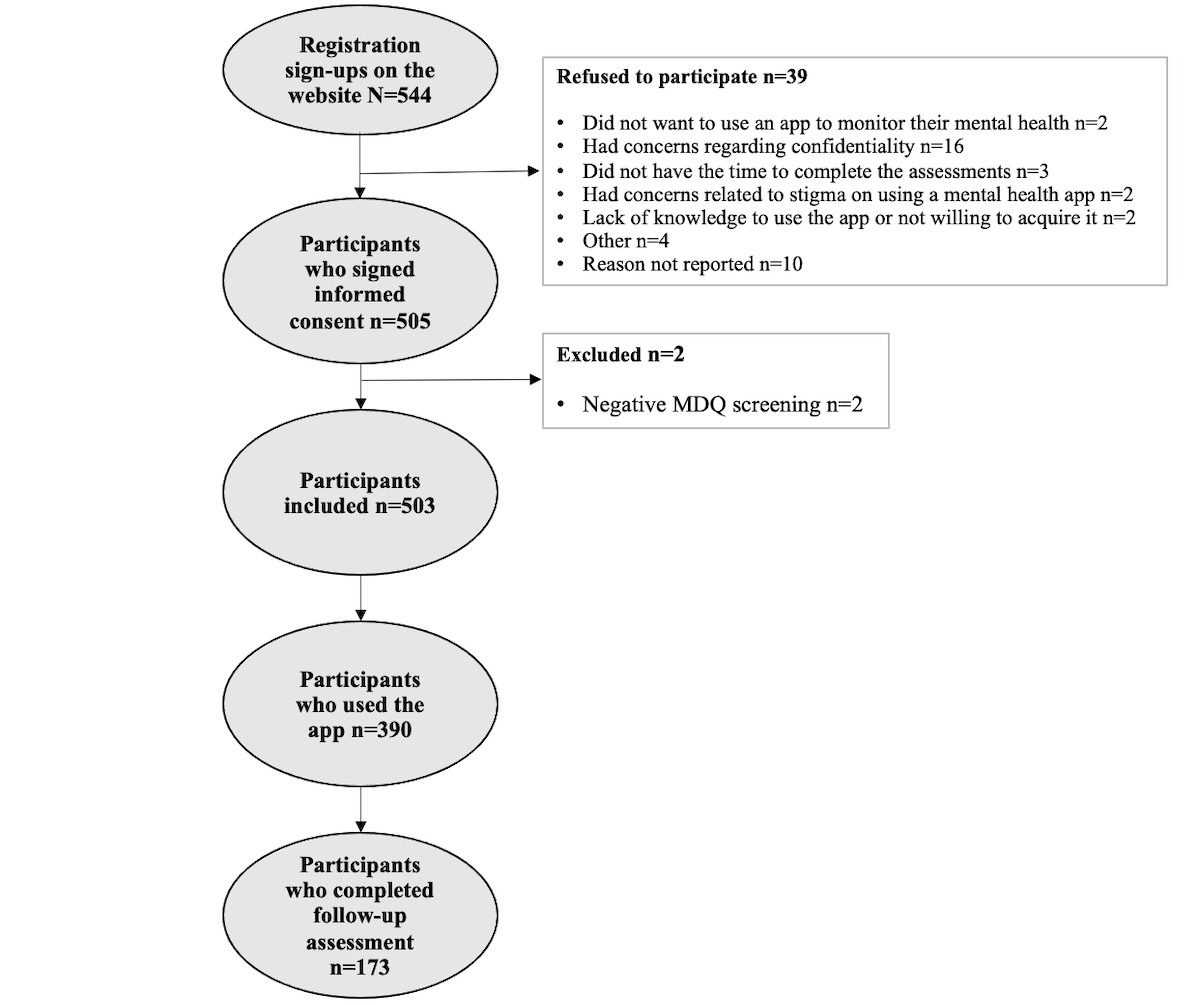
Flowchart of participants included in the statistical analysis. MDQ: Mood Disorder Questionnaire.

Figure 1 depicts the number of participants registered on the OpenSIMPLe website who were initially interested in using the app alongside those included in the statistical analysis. The reasons reported by users who did not want to participate in the study are also listed. Finally, potential participants who were excluded are described, as well as the number of participants who actually used the app and those who responded to the 6-month follow-up assessment.

### Sociodemographic and Clinical Characteristics

The mean age of users was 34.74 (SD 10.48) years, and most (264/390, 67.7%) of them were women. The most frequent ethnicity was Latin American (266/390, 68.2%), with high education levels (241/390, 61.8%). A high percentage of the sample was employed at the time of study entry (156/390, 40%), whereas only 17.7% (69/390) were either on temporary or permanent disability leave. Regarding housing conditions, 33.8% (132/390) of the participants lived in the parental household and more than half of the sample reported living independently, either owning (117/390, 30%) or renting their current house or flat (90/390, 23.1%). Sociodemographic variables of the SIMPLe app users are described in Table 1.

Regarding the clinical variables, a mean disorder duration of 13.23 (SD 9.97) years was identified; 49.7% (194/390) of the users stated that they had experienced ≥10 depressive episodes; and 33.3% (130/390) reported ≥10 manic or hypomanic episodes. Most of the participants who used the app were receiving treatment with at least one mood stabilizer (353/390, 90.5%) and at least one antipsychotic (252/390, 64.6%), whereas almost half (193/390, 49.5%) of the participants were receiving at least one antidepressant. Furthermore, 71.5% (279/390) of the participants were receiving some kind of psychological treatment. The clinical variables collected at baseline are described in Table 2.

**Table 1:** Baseline sociodemographic characteristics of participants (N=390).

Characteristic		Value
Gender, female, n (%)		264 (67.7)
**Ethnicity, n (%)**		
	African	2 (0.5)
	White	119 (30.5)
	Latin American	266 (68.2)
	Asian	2 (0.5)
	Other	1 (0.3)
Age (years), mean (SD)		34.74 (10.48)
**Marital status, n (%)**		
	Single	192 (49.2)
	Married	81 (20.8)
	Cohabitation	50 (12.8)
	Divorced or separated	54 (13.8)
	Widowed	1 (0.3)
	Other	12 (3.1)
**Housing status, n (%)**		
	Shared home	43 (11)
	Tenant	90 (23.1)
	Homeowner	117 (30)
	Parental home	132 (33.8)
	Residence or institution	8 (2.1)
**Completed studies, n (%)**		
	None	1 (0.3)
	Primary education	8 (2.1)
	Secondary education	67 (17.2)
	A-level or general certificate of education	73 (18.7)
	Vocational education and training or certificate of higher education or higher national diploma	95 (24.4)
	Bachelor’s degree	101 (25.9)
	Graduate certificate or postgraduate diploma or master’s degree	45 (11.5)
**Employment status, n (%)**		
	Unemployed	78 (20)
	Student	81 (20.8)
	Employed	156 (40)
	Retired	10 (2.6)
	Temporary disability leave	35 (9)
	Permanent disability leave	30 (7.7)
**Country, n (%)**		
	Spain	130 (33.3)
	Chile	76 (19.5)
	Argentina	66 (16.9)
	Mexico	25 (6.4)
	Colombia	23 (5.9)
	Guatemala	12 (3.1)
	Brazil	9 (2.3)
	Other	49 (12.6)

**Table 2:** Baseline clinical variables of app users (N=390).

Illness course			Value
Years since onset, mean (SD)			13.23 (9.97)
Years since diagnosis of bipolar disorder, mean (SD)			6.4 (6.55)
**Depressive episodes, n (%)**			
	0-4	110 (28.2)	
	5-9	86 (22.1)	
	≥10	194 (49.7)	
**Manic or hypomanic episodes, n (%)**			
	0-4	143 (36.7)	
	5-9	117 (30)	
	≥10	130 (33.3)	
**Previous hospital admissions because of an episode, n (%)**			
	None	185 (47.4)	
	1-2	135 (34.6)	
	≥2	70 (17.9)	
**Suicide attempts, n (%)**			
	None	156 (40)	
	1-2	142 (36.4)	
	≥2	92 (23.6)	
**Treatment setting, n (%)**			
	Public health network	145 (37.2)	
	Private health network	184 (47.2)	
	Both	61 (15.6)	
**Past psychological treatment, n (%)**			
	None	39 (10)	
	Yes, individual psychotherapy	260 (66.7)	
	Yes, group psychotherapy	9 (2.3)	
	Yes, individual and group psychotherapy	82 (21)	
**Current psychological treatment, n (%)**			
	None	111 (28.5)	
	Individual psychotherapy	230 (59)	
	Group psychotherapy	15 (3.8)	
	Individual and group psychotherapy	34 (8.7)	
**Current pharmacological treatment, n (%)**			
	Mood stabilizer	353 (90.5)	
	Antipsychotic	252 (64.6)	
	Antidepressant	193 (49.5)	
	Anxiolytic	183 (46.9)	

### Engagement

The 503 participants included in the study can be divided into three broad categories based on their app use: no users (never used the app), occasional users (engagement <12%), and regular users (engagement ≥12%). Of the 503 participants, 113 (22.5%) were no users, 357 (70.9%) were regular users, and 33 (6.6%) were occasional users. In addition, among the participants who used the app, 44.4% (173/390) completed the follow-up assessment too.

We analyzed the number of days containing any kind of record in the app from users over the 6-month follow-up period. Monthly progress of regular users’ engagement declined gradually over the first 6 months. The highest engagement was observed in the first month (mean 0.74, SD 0.20); in the second month, it dropped sharply. At 6 months, the users had a mean engagement of 0.39 (SD 0.34).

Occasional users used the app a mean of 139.06 (SD 56.80) days, with a use frequency of 2.05 (SD 0.87) days per month, whereas regular users used the app 83.98 (SD 69.95) days, with a use frequency of 19.42 (SD 7.76) days per month. The group of occasional users rarely used the app over long periods of time, which could overestimate use time rates, for example, a participant who used the app at the 3-month follow-up for the last time but only used the app 4 times overall. The estimate that this participant used the app for 3 months may lead us to a use bias. As the SIMPLe app was developed for daily use, we could consider that the interaction of this kind of user with the app is low enough to overestimate the time of use in the statistical analysis. To avoid this bias, we used the variable overall engagement—the percentage of tasks completed compared with those expected to be completed during the time in which they used the app—to identify users who may make us enhance the time-of-use overestimation. Occasional users were ruled out in the survival analysis.

Sensitivity and homogeneity analyses show homogeneous baseline variables across subsets of participants defined by retention duration, with the exception of the *cocaine use* variable, which was significantly greater among the group of no users than among the occasional and regular users. In addition, we performed survival analysis again with all users to assess the effect of the selection of regular users in the estimations of retention duration. The results showed a 7-day increase in the median survival time when we included occasional users in the analysis, whereas the probability of survival only increased by a score of 0.027 (SD 0.007) on average. Therefore, we are confident that selection bias did not occur and that the subsets were representative of the data of the sample.

### Use and Retention Duration

We analyzed the number of days with records in the app from users over the 6-month follow-up period. It turned out that only 13.8% (54/390) of the users used the SIMPLe app for >100 days. The mean survival time of regular app users was 87.95 (SD 72.08; 95% CI 80.48-95.43) days.

The probability of survival for the 357 participants under consideration for these analyses after 1 month of app use was 67.4% (95% CI 62.7%-72.4%); at 3 months, the probability of survival was 43% (95% CI 38.1%-48.5%); and at the 6-month follow-up assessment, the probability of survival was 28% (95% CI 23.6%-33.2%). The risk of discontinuing app use increased as the days passed: at 3 months, the cumulative risk of discontinuing app use was 83.7%; however, at 6 months, this rose to a cumulative risk of 126.3%.

**Figure 2:**
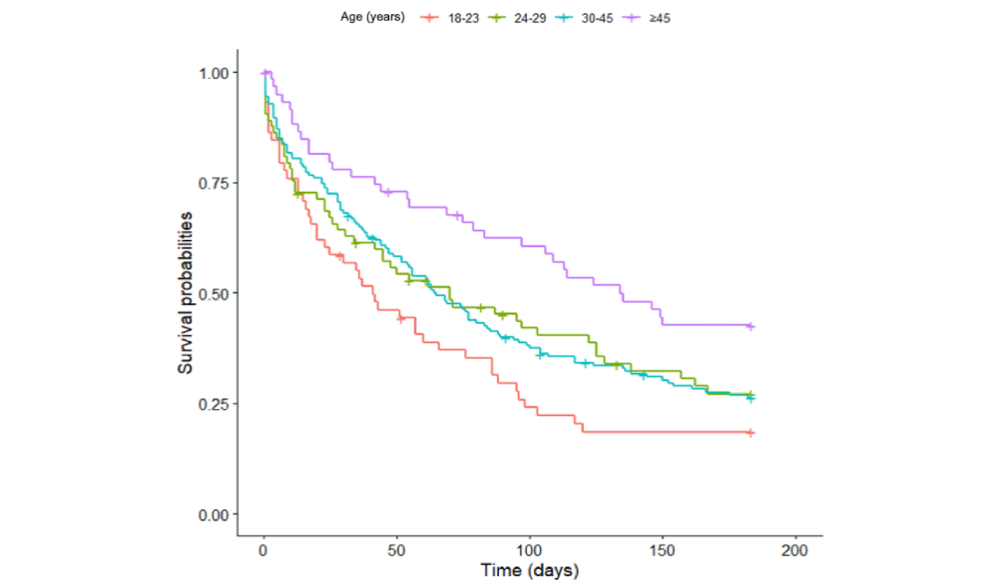
Plot of Kaplan–Meier age estimates of survival of participants using the SIMPLe app. The horizontal axis represents the survival time (in days) with records in the app (6 months maximum).

The correlations between retention duration and the sociodemographic and clinical variables of users at baseline were analyzed. A direct correlation between age and engagement was observed (*ρ*=0.168; *P*=.002); older users (aged ≥46 years) had higher total app use (mean 109.78, SD 71.42; *P*=.005) than younger users (aged 18-23 years; mean 63.12, SD 63.94; *P*=.005; Figure 2).

Time passed since illness onset was strongly associated with time of use (*ρ*=0.194; *P*<.001) and years since diagnosis of BD (*ρ*=0.106; *P*=.048). In addition, participants who had been diagnosed with BD for longer (>17 years) used the app for more days (mean 97.73, SD 69.15; *P*=.002) than those who had had a more recent onset (mean 66.49, SD 66.18; *P*=.002) or those who had been diagnosed <6.5 years ago (mean 73.45, SD 66; *P*=.01).

We performed log-rank (Mantel–Cox) test analysis to compare survival curves and detect potential factors contributing to the risk of discontinuing app use (Table 3). Variables with a significant contribution were years since onset (*χ*^2^_2_=11.7; *P*=.003), years with BD diagnosis (*χ*^2^_1_=8.9; *P*=.003), opiate use (*χ*^2^_1_=7.9; *P*=.005), age (*χ*^2^_3_=12.3; *P*=.006), housing status (*χ*^2^_4_=12.3; *P*=.006), employment status (*χ*^2^_5_=13.5; *P*=.02), and antipsychotic use (*χ*^2^_1_=4.9; *P*=.03).

Survival-curve plots for the variables of interest were produced over the 6-month-long follow-up period and are described in the following paragraphs (Table 4).

**Table 3:** Log-rank test for overall survival^a^.

Variables			Log rank (Mantel–Cox)		
			Chi-square (*df*)		*P* value
**Clinical variables**					
	Comorbid psychiatric disorder	2.5 (1)		.11	
	Anxiety disorder	3.9 (1)		.04	
	Personality disorder	2.4 (1)		.12	
	Substance abuse disorder	0.5 (1)		.46	
	Eating disorder	2.0 (1)		.15	
	PTSD^b^	1.1 (1)		.30	
	Other comorbid psychiatric disorders	2.2 (1)		.13	
	WHO-5^c^	2.9 (2)		.23	
**Illness course**					
	Years since onset of first episode	11.7 (2)		.003	
	Years diagnosed with bipolar disorder	8.9 (1)		.003	
	Depressive episodes	4.2 (2)		.12	
	Manic or hypomanic episodes	4.5 (2)		.10	
	Hospitalizations because of an episode	2.1 (2)		.35	
	Suicide attempts	1.9 (2)		.36	
**Treatment**					
	Psychotherapy	1.1 (3)		.77	
	Mood stabilizer	0.2 (1)		.65	
	Antipsychotic	4.9 (1)		.02	
	Antidepressant	0.0 (1)		.84	
	Anxiolytic	0.1 (1)		.73	
	Electroconvulsive therapy	1.8 (1)		.17	

^a^Users with engagement ≥12%.

^b^PTSD: posttraumatic stress disorder.

^c^WHO-5: 5-item World Health Organization Well-being Index.

**Table 4:** Comparing survival curves.

Characteristic			Regular users^a^		
			Mean, estimate (SE; 95% CI)		Median, estimate (SE; 95% CI)
**Age (years)**					
	18-23	65.72 (8.60; 48.85-82.58)		41.00 (9.90; 21.59-60.40)	
	24-29	87.11 (8.69; 70.06-104.16)		70.00 (23.12; 24.67-115.32)	
	30-45	86.26 (5.54; 75.40-97.12)		65.00 (7.28; 50.72-79.27)	
	≥46	114.83 (9.17; 96.85-132.80)		134.00 (22.24; 90.39-177.60)	
**Housing status**					
	Shared home	62.65 (9.91; 43.21-82.08)		50.00 (8.51; 33.31-66.68)	
	Tenant	98.03 (7.71; 82.91-113.15)		83.00 (16.02; 51.58-114.41)	
	Homeowner	99.95 (7.34; 85.56-114.34)		109.00 (26.39; 57.26-160.73)	
	Parental house	77.52 (6.35; 65.05-89.98)		56.00 (10.86; 34.70-77.29)	
	Residence or institution	96.62 (20.35; 56.72-136.52)		62.00 (60.81; 0.00-181.19)	
**Employment status**					
	Unemployed	72.87 (7.74; 57.69-88.05)		55.00 (12.96; 29.58-80.41)	
	Student	75.02 (7.85; 59.62-90.42)		57.00 (14.14; 29.28-84.71)	
	Employed	97.40 (6.09; 85.45-109.36)		80.00 (12.27; 55.9-104.06)	
	Retired	109.40 (24.72; 60.93-157.86)		84.00^b^	
	Temporary disability leave	101.96 (13.21; 76.06-127.86)		104.00 (61.23; 0.00-224.01)	
	Permanent disability leave	85.14 (14.47; 56.77-113.52)		69.00 (24.71; 20.55-117.44)	
**Diagnostic time (years)**					
	0-6.5	81.14 (4.79; 71.74-90.54)		61.00 (7.45; 46.38-75.61)	
	>6.5	98.15 (6.36; 85.67-110.63)		94.00 (15.98; 62.67-125.32)	
**Anxiety disorder**					
	No	95.07 (4.91; 85.43-104.71)		83.00 (9.17; 65.01-100.98)	
	Yes	78.19 (5.93; 66.56-89.83)		46.00 (7.96; 30.39-61.61)	

^a^Users with engagement ≥12%.

^b^SE and 95% CI are not available.

The app use survivorship of the oldest participants (aged ≥46 years) seems greater than that of the youngest group of users (aged 18-23 years) because the estimated mean was 114.83 (95% CI 96.85-132.80) days for users in the former age range, whereas the use mean was 65.72 (95% CI 48.85-82.58) days for users in the latter group. At 60 days, the probability of survival of the youngest users was 38.9% (95% CI 28%-53.9%); this likelihood increased to 69.4% for the oldest group of participants, with a cumulative risk of 0.923 and 0.36, respectively.

Regarding housing status, we observed that the mean estimation of app use survival of people sharing a house or flat was 62.65 (95% CI 43.21-82.08) days, that is, between 15 and 37 days fewer than users with other housing statuses, suggesting a survival disadvantage. At 60 days, the cumulative incidence risk estimates among users who shared a house were 0.880, whereas they were 0.435 for individuals who lived in residences, 0.488 for tenants, 0.509 for homeowners, and 0.734 for those who lived in the parental home.

Being unemployed seemed to worsen app use survivorship pretty much at all time points because the survival likelihood mean estimation of unemployed participants was 72.87 (95% CI 57.69-88.05) days, that is, lower than any other employment status. At 60 days, the highest survival cumulative risk was that of unemployed participants (0.737), followed by students (0.734), individuals on permanent (0.596) and temporary disability leave (0.573), employers (0.523), and retired people (0.336).

In addition, app use declined faster among participants who had been recently diagnosed (<6.5 years) compared with users who had been diagnosed for a longer period of time; at 60 days, the cumulative risk of app use discontinuation among people who had a recent diagnosis was 0.681, whereas this risk was lower for those who had been diagnosed earlier (0.518). The mean app use estimation of individuals with a more recent diagnosis of BD was 8.14 (95% CI 71.74-90.54) days, whereas it increased to 98.15 (95% CI 85.67-110.63) days for people with an earlier diagnosis.

The survival time of patients with comorbid anxiety disorder diverged from those who did not have symptoms of it over time, with a cumulative risk of use discontinuation of 0.789 and 0.500 at 60 days, respectively. Relatively few patients continued to use the app after the very first month overall, but among those who were still using it, participants with anxiety disorder continued to show a survival disadvantage over those who did not experience it. The mean estimation of app use of the latter group was 95.07 (95% CI 85.43-104.71) days, whereas that of participants with an anxiety disorder was 78.19 (95% CI 66.56-89.83) days; therefore, having an anxiety disorder significantly influenced app use. Nevertheless, anxiety disorder was self-reported based on what users consider anxiety; hence, we tried to see if there was homogeneity between self-reports and treatment prescriptions at study initiation. Analysis showed that there is a statistically significant relationship between anxiolytics use and self-reported anxiety disorder (*P*<.001), and 59.6% (99/166) of the users who reported having an anxiety disorder did use them, whereas anxiolytics use decreased to 37.5% (84/224) among the participants who did not report an anxiety disorder. As antidepressant drugs can also be used to treat a number of other conditions, including anxiety disorders, we also analyzed association of self-reported anxiety disorder with anxiolytics, along with the most widely prescribed antidepressants for anxiety. It turned out that 74.7% (124/166) of the users who reported having an anxiety disorder did use these medications, despite the guideline recommendations to avoid antidepressants in BD.

Furthermore, we performed a Cox (proportional hazards) regression analysis to estimate the hazard of discontinuing app use for regular users, given their prognostic variables. The results of the Cox model analysis are presented in Table 5.

**Table 5:** Cox regression model analysis of user survival using the SIMPLe app 1.5^a^.

Characteristics		Coefficient	Exp (coefficient; 95% CI)	*P* value	Concordance, mean (SE)
Age		–0.016	0.984 (0.971-0.998)	.02	0.589 (–0.019)
**Country**					N/A^b^
	Spain	—^c^	—	—	
	Chile	–0.143	0.867 (0.588-1.278)	.47	
	Argentina	0.091	1.096 (0.748-1.605)	.63	
	Mexico	–0.121	0.886 (0.495-1.585)	.68	
	Colombia	0.318	1.375 (0.797-2.37)	.25	
	Other	0.141	1.152 (0.779-1.703)	.47	
Anxiety disorder		Yes	0.233	1.262 (0.975-1.634)	.07
Antipsychotic		Yes	0.334	1.396 (1.058-1.843)	.02

^a^Schoenfeld residuals to check the proportional-hazards assumption: age (*χ*^2^_1_=0.006; *P*=.94), anxiety disorder (*χ*^2^_1_=2.7; *P*=.10), and antipsychotic (*χ*^2^_1_=0.1; *P*=.72).

^b^N/A: not applicable.

^c^Our Cox model analyzed the risk of discontinuing the app use that participants from different nationalities had in comparison with Spanish participants. This row was maintained in the table to make clear that Spain was not included in the category *Other*.

The variables age, anxiety disorder, antipsychotic, and country were explored (Table 5). In this model, age is suggested as a protective factor for app use discontinuation, that is, the older the individual, the lower the risk of discontinuing app use. The regression coefficient for age was –0.016 (*P*=.02), which would imply a better engagement for older individuals. We calculated the percentage change in hazard rate for years’ increase in age using the formula 100 × (e^(–0.016×10)^ – 1) = –14.8, which allows us to estimate that a user older by 10 years would have a 14.8% reduction in their hazard compared with a user younger by 10 years.

We did not observe statistically significant differences among countries. Participants from the countries analyzed did not have a significantly different risk of discontinuing app use compared with Spanish users.

The regression coefficient for taking antipsychotics is statistically significant (coefficient=0.334, 95% CI 1.058-1.843; *P*=.02), which suggests that this variable is a risk factor and that users who take antipsychotics have a 33% hazard for discontinuing app use.

### Usability, Satisfaction, and Perceived Usefulness

The analysis of user evaluation of the SIMPLe app contained in this section was exclusively performed with data of the 173 participants who used the app and completed the follow-up assessment too.

**Figure 3:**
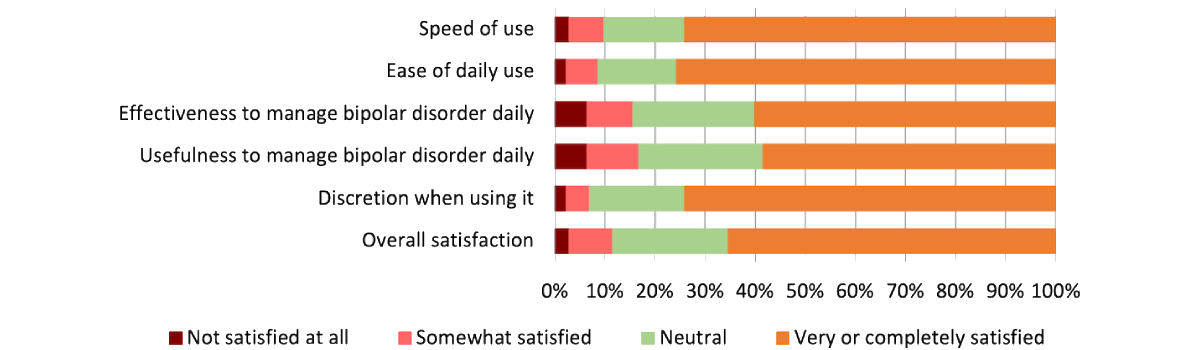
Satisfaction with the SIMPLe app. The bars denote the percentage of satisfaction of users who responded to the follow-up questionnaire (n=173) after having experienced using the app.

The mean raw SUS score was 77.05 (SD 17.21), which is above average at the 75th percentile. As shown in Figure 3, 74% (128/173) of the users were highly or completely satisfied with the speed and discretion of the app and 75.7% (131/173) of the users were satisfied with the ease of daily use. A high or complete level of overall satisfaction was rated by 65.3% (113/173) of the users.

With regard to usefulness, most users found somewhat useful or very useful the following features and functions: daily test (125/173, 72.3%), mood chart (127/173, 73.4%), personalized psychoeducational messages (117/173, 67.6%), weekly test (117/173, 67.6%), stressful events record (108/173, 62.4%), emergency alert notifications (103/173, 59.6%), the enabled option to share the mood chart (94/173, 54.3%), and the prodromes module (87/173, 50.3%).

Among the users who registered the reason for discontinuing using the app (91/173, 52.6%) by answering a multiple-choice question, 28.6% (26/91) found it very repetitive, 23.1% (21/91) had technical issues, 17.6% (16/91) did not find it useful, 16.5% (15/91) stated that it was an undesired daily reminder of their condition, 14.3% (13/91) stated that it affected smartphone performance, 13.2% (12/91) gave other reasons, 7.7% (7/91) were concerned about the stigma attached to having it installed on their smartphone, 4.4% (4/91) relapsed, and 3.3% (3/91) found it difficult to use.

Users suggested app improvements by responding to a multiple-choice question. The improvements more frequently suggested were enabling a personalized plan to follow when potential relapse symptoms appear (122/173, 70.5%), personalization of stressful events (103/173, 59.5%), and a wider variety of psychoeducational messages (99/173, 57.2%).

## Discussion

After proving positive outcomes regarding satisfaction, usability, and helpfulness in previous research, the SIMPLe app was launched to the real world to make it available worldwide within the context of BD treatment.

### Principal Findings

The outcomes of this real-world study represent the first attempt to evaluate, by means of survival analysis, use, retention patterns, and engagement of a large-scale wide-reaching app-based intervention providing psychoeducational content to patients with BD.

One of the most important advantages of the data collected through smartphone apps in clinical studies is the continuous and granular characteristics of the data registered at servers. Using detailed log and use data to examine predictive factors allows an understanding of the engagement and its underlying mechanisms. It will also aid optimization of smartphone-based interventions and improvement in the real-world uptake of self-management apps for BD, as well as in clinical benefits and associated outcomes [33].

### Comparison With Prior Work

Survival analysis is not a new idea in statistics, and it is frequently used in several medical fields; in fact, it was considered the main outcome measure in the seminal works by Colom [34], who first showed the efficacy of group psychoeducation of patients in participants with BD. However, this method is a novel approach to quantify user retention and engagement of mobile apps that few studies have previously applied in nonpsychiatric populations [35,36]. Interestingly, Chien et al [37] used machine learning techniques to identify heterogeneity in patient engagement with internet-based cognitive behavioral therapy (CBT) for symptoms of depression and anxiety and found that patterns of patient behavior may reveal different modalities of engagement.

The aforementioned results are in line with one of the largest data sets on engagement in remote digital health compiled to date [38] (there is significant attrition in remote research). In addition, Pratap et al [38] observed indicators of retention in remote digital health studies through survival analysis, which revealed the factors associated with an increase in participant retention time, including older age (an increase of 4 days). In contrast, our study revealed that years since onset and years since diagnosis of BD (2 variables highly related to age) had a significant impact on app use; mobile app median use of participants with earlier disease onset and diagnosis increased by 32 days and 29 days compared with individuals with a more recent onset and more recent diagnosis, respectively. Similarly, when comparing potential predictors of traditional group psychoeducation in BD in a digital format, Reinares et al [39] identified that receiving an early diagnosis of BD may indicate a better response to face-to-face group psychoeducation. Other factors with a significant contribution to the risk of discontinuing app use (that are also highly associated with age) were housing and employment status. Retention duration of unemployed participants and those who shared a house was lower than that of users with other housing statuses. It is obvious that having a recent onset and diagnosis, sharing a house, and unemployment are more common in younger populations, suggesting a disadvantage regarding app use.

Our findings are consistent with previously collected preliminary use data on the SIMPLe app [22]. We previously observed that older age was a predictor significantly associated with higher odds of retention. In addition, the attrition rate of the program was still high, but this time our research focused on retention factors. Furthermore, overall satisfaction of the participants was quite positive because 65.3% (113/173) were highly or completely satisfied with SIMPLe in the context of low retention. The satisfaction, usability, and helpfulness outcomes are in line with our previous results deriving from an implementation feasibility study of SIMPLe. In terms of usefulness, the best-rated features and functions of the app were daily and weekly tests, mood charts, and personalized psychoeducational messages. The last-named is a unique and differentiating feature that other mobile-based platforms for BD do not offer [40]. On the basis of the information collected through screening tests on mood, energy, sleep time, medication adherence, and irritability, the SIMPLe app pushes daily pop-up notifications with a short psychoeducational message containing brief information (usually fewer than 50 words) on how to deal with specific situations to avoid relapses. Psychoeducational short messages carefully provided to cover detected specific user needs are the closest way to feature the *human touch*, and they fulfill quite convincingly our initial intention to partly replicate the successful Barcelona Bipolar Psychoeducation model [18] in an app so that it could de-escalate treatment costs and make combined therapy (psychoeducation and psychopharmacology) available to the greatest number of affected individuals.

In contrast, Faurholt-Jepsen et al [41] investigated the effect of smartphone-based patient-reported and objective monitoring, including a mood prediction system. The collected objective smartphone data included phone use, social activity, and mobility. Patients with BD were randomized to the Monsenso monitoring system (Monsenso A/S) or to standard treatment. Clinical feedback was established for patients in the intervention group in the case of signs of impairment (eg, lack of self-monitoring data). Overall, there was no effect of smartphone-based monitoring on symptoms compared with the control group. The intervention group adhered to the daily self-monitoring 72.6% (196/270) of the days over the 9-month study period.

Yet another project [42] evaluated 2 mobile phone–augmented interventions: an in-person session of CBT combined with automated thought-challenging and adaptive behavior delivered through mobile devices and an in-person session of psychoeducation with mobile interaction involving self-monitoring of symptoms. The retention rates were 77% for the self-monitoring group and 91% for the CBT condition at 6 months. Follow-up in both active conditions consisted of telephone calls by the study therapist to remind participants of assessment appointments and encourage adherence. However, these outcomes are difficult to extrapolate and compare because the cited Californian study included patients with schizophrenia as well as patients with BD. Previously, Depp et al [43] had explored a similar approach integrating a mobile device–delivered intervention linking patient-reported mood states with self-management strategies, preceded by face-to-face brief psychoeducation in BD. At 12 weeks, the retention reported was 93%; however, it was only operationalized based on the percentage of participants who returned the borrowed mobile devices of the study.

At odds with the aforementioned studies, the SIMPLe app was designed as an independent self-management method targeting relapse prevention. For research purposes, the study team helped (remotely) participants to install the app and log into the system, as well as provided a brief explanation. Users were provided with a telephone number to contact the research team for further assistance in case they experienced technical issues. The retention rate of the original SIMPLe study was 74% (36/51) after 3 months of app use [21].

The OpenSIMPLe study differs from the others in that it is the only modality that does not involve some contact with a person providing support or human interaction. It is reasonable to assume that the lower retention rates of the OpenSIMPLe study may have been influenced by the absence of human support in comparison with the other studies assessing smartphone-based platforms; the latter were more demanding in terms of time and staff resources. Besides the notifications systems recalling adherence in the aforementioned studies, the fact that participants established an alliance with clinical study staff and received previous face-to-face intervention or continuous telephone-delivered psychoeducation has influenced retention for certain [44].

It has been suggested that a positive relationship between app engagement and improvements in therapeutic outcomes in mental health and well-being may indicate the effectiveness of internet-based interventions [45]. Moreover, the type of engagement in terms of intervention use has demonstrated different relationships with outcomes [46]. However, there is a general tendency among mental health apps toward low retention and engagement rates [32,47]. At the same time, detailed engagement and retention rates are rarely reported in smartphone-based clinical trials but are of crucial importance to understand the underpinning of their effects. Those studies reporting and analyzing them frequently do so with heterogeneous and nonstandardized methodologies.

It is worth mentioning that we are comparing our retention, app use, and engagement data with other studies that used different parameters to measure these indicators for mental health apps and even used different criteria to assess them. Results from a systematic review conducted by Ng et al [32] indicated high variability across studies regarding the operationalization of the indicators of engagement, as well as highlighted the need to using objective criteria when assessing them. To date, the lack of clear consensus on the definition and standardization of these parameters represents a big obstacle to understanding feasibility and comparing results in the field of mobile-based apps and digital therapeutics. The real effectiveness of every intervention can be hardly estimated without quantifying the exact *dosage* of intervention that individuals receive, and engagement is not usually considered or it is evaluated erratically through effect size estimation in randomized controlled trials.

### Limitations

Some limitations of this study should be noted, and caution should be exercised in generalizing results. First, our analyses relied on a rather heterogeneous sample, where participants differed in terms of sociodemographic, clinical, and psychological characteristics. This can be explained by the fact that we opened the platform to a real-world setting without considering inclusion and exclusion criteria that were too restrictive. However, the participants were a good representation of an unselected real-world population.

All measures were administered using exclusively self-reported web-based methods that did not allow us to get back in touch with participants who dropped out to collect feedback on the reasons for attrition. In addition, we did not have access to either medical records or passive data to validate the accuracy and reliability of the information provided, which may have influenced our sample and outcomes. As shown in Figure 1, there were only 2 participants excluded by the MDQ at screening. The MDQ is a popular, simple, and sensible screening instrument for the detection of BD. However, this tool is far from perfect [48]; it has low specificity, and it is likely that it did not discriminate among participants with a range of disorders such as borderline personality disorder. This is a common disadvantage in studies that screen participants through web-based methods exclusively.

A weakness of this study is that we limited use and retention analysis to the regular users of the SIMPLe 1.5 app and did not analyze other data from the occasional users, who did not use the app consistently. However, the aim of limiting these analyses to data provided by regular users was to avoid overestimation of use time and retention. In addition, sensitivity and homogeneity analysis confirmed that the data were coherent when we repeated the survival analysis with the whole sample of users; this showed that the selection of regular users in the study of app use prevented an overestimation of it, whereas the effect of the selection on survival probability is small.

Furthermore, only 44.4% (173/390) of the users completed the follow-up assessment, which implies some bias in the data collected regarding evaluation of the app because the variables measured at follow-up (including SUS, perceived usefulness, and satisfaction) were exclusively assessed by these users. An example that indicates this bias is that we found differences in the time range of app use between patients who used the SIMPLe app (n=390) and those who used it and completed the follow-up assessment (n=173); the former group used it a mean of 88.64 (SD 70.56) days, whereas this mean increased to 119.64 (SD 69.9) days in the latter group. However, the former group used the app slightly more every day on average (mean 0.92, SD 0.73) than the latter group (mean 0.83, SD 0.51). Therefore, these differences in terms of app use between the groups suggest that the users may have different profiles.

As this work was an exploratory study (ie, a flexible rather than structured approach to data collection was considered useful), there was no control group or alternative intervention for comparison of effects because the study was not designed to test the efficacy of the SIMPLe app. For the same reason and to avoid unlimited assessments that would probably result in the attrition rate soaring to unacceptable levels, we decided to keep control and covariate data to a minimum, which obviously represents at the same time something gained and something lost.

All the participants included came from Latin American or Spanish populations. The cultural characteristics of these origins may be difficult to generalize, but little is known about app adherence (or even drug or psychotherapy adherence) across cultures. This may become an exciting topic awaiting proper exploration.

Finally, it should be considered that the outcomes of this study deal with a high level of missing data derived from highly variable retention rates and lack of adherence after a few weeks of use among users of mental health apps, which is a common hindrance in internet-based research [12,49] that we tried to handle in an honest and rigorous manner.

### Conclusions

The user retention rate of the app decreased at a rapid rate after each month until reaching only one-third of the users at 6 months. There exists a strong association between age and app engagement of individuals with BD. Other variables such as years lived with BD, diagnosis of an anxiety disorder, and taking antipsychotics seem to play a relevant role as well. We believe that an understanding of these associations will help clinicians in the definition of the most suitable user profiles for predicting trends of engagement, optimization of app prescription, and management.

## Abbreviations

BD: bipolar disorder
CBT: cognitive behavioral therapy
MDQ: Mood Disorder Questionnaire
SUS: System Usability Scale
